# Vanishing Lung Syndrome: A Case Report and Systematic Review of the Literature

**DOI:** 10.7759/cureus.53443

**Published:** 2024-02-02

**Authors:** Meghan Mansour, Steven Kessler, Ali Khreisat, Jacob Morton, Ramona Berghea

**Affiliations:** 1 Internal Medicine, Oakland University William Beaumont School of Medicine, Rochester Hills, USA; 2 Internal Medicine, Corewell Health William Beaumont University Hospital, Royal Oak, USA

**Keywords:** pneumothorax, emphysema, copd, giant pulmonary bullae, acute vanishing lung syndrome

## Abstract

Vanishing lung syndrome (VLS), also known as idiopathic giant bullous emphysema, is defined by the emergence of sizable bullae causing compression on healthy lung tissue. The elusive etiology of VLS mandates a diagnosis based on radiographic evidence showcasing giant bullae occupying at least one-third of the hemithorax in one or both lungs. This report presents a case of VLS in a 36-year-old female smoker devoid of any prior medical history. Additionally, we conducted a systematic review to discern the demographics, risk factors, and treatment modalities for individuals diagnosed with VLS.

## Introduction

Vanishing lung syndrome (VLS), or idiopathic giant bullous emphysema, is characterized by the development of large bullae that compress healthy lung tissue [1]. Diagnosis includes radiographic evidence of giant bullae in one or both lungs, occupying at least one-third of the hemithorax [2]. The etiology of VLS is elusive, yet family history, smoking, cannabis use, and alpha-1-antitrypsin deficiency may be risk factors [3-5]. Patients with VLS can be asymptomatic or can have a broad range of symptoms including progressive shortness of breath, productive cough, functional decline in performance status, and occasionally nontraumatic pneumothorax [5]. Giant emphysematous lung bullae develop from inflammatory destruction and loss of elasticity of small alveolar walls which then coalesce into large air-filled bullae [6]. Complications of VLS include respiratory failure, spontaneous pneumothorax, pneumonia, or compression of surrounding mediastinal structures [5,7]. We report a case of VLS in a 36-year-old female smoker without any previous medical history. We also conducted a systematic review to identify the demographics, risk factors, and treatments for patients with VLS.

## Case presentation

**Figure 1:**
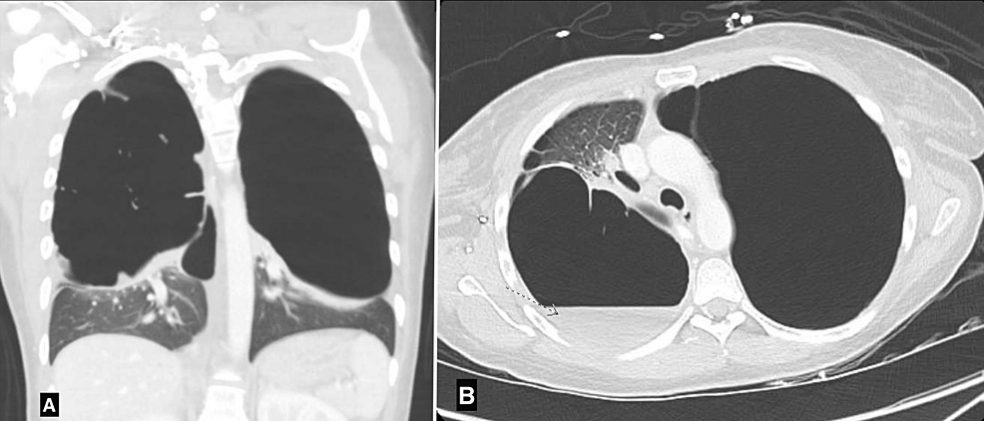
Chest CT scan coronal view (A) and transverse view (B) showing severe bilateral apical bullous emphysema without pneumothorax and an air-fluid level in the right apical bullae (arrow) suggestive of superimposed pneumonia CT: computed tomography

A 36-year-old female with an unremarkable medical history presented with right-sided chest pain, dyspnea, and intermittent productive cough persisting for four months. She also reported rhinorrhea and unintentional weight loss over the past year. Her social history included a 16-year history of daily smoking (four cigarettes per day) and cannabis use. An initial chest X-ray suggested a possible pneumothorax. Coronal (Figure 1a) and apical (Figure 1b) computed tomography (CT) scans showed severe bilateral apical bullous emphysema without pneumothorax or pulmonary embolism and an air-fluid level in the right apical bullae suggestive of superimposed pneumonia. Coronavirus disease 2019 (COVID-19) infection was confirmed. Inhaled bronchodilators, inhaled steroids, prednisone, and azithromycin for COVID-19-related chronic obstructive pulmonary disease (COPD) exacerbation were initiated.

Further testing revealed normal alpha-1-antitrypsin levels and negative autoimmune markers. Human immunodeficiency virus (HIV) and syphilis testing were negative. VLS became a leading consideration. The patient's chest pain improved, and she was discharged to follow up with thoracic surgery. One month later, she was readmitted with chest pain. Outpatient pulmonary function tests (PFTs) showed forced expiratory volume in one second (FEV_1_) at 48% of predicted (Table 1), diffusion capacity of the lungs for carbon monoxide at 45% of predicted, and severe air trapping with a residual volume of 279% of predicted for her age (Table 2). A CT chest scan revealed the development of an air-fluid level within a right posterior bulla, likely representing hemorrhage or infection, and an increase in the size of her bi-apical bullae. Empiric antibiotic therapy was administered. With the diagnosis of VLS still in question, she was again discharged.

**Table 1:** The patient's spirometry showing severe obstructive airway disease. FEV1 is only 36% of predicted with a good response to inhaled bronchodilators FEV_1_: forced expiratory volume in one second; FVC: forced vital capacity; FEF: forced mid-expiratory flow; PEF: peak expiratory flow

Spirometry	Predicted (reference)	Best	%Predicted	Post bronchodilator	Post-predicted%	%Change
FVC (L)	2.98	1.43	48%	1.72	58%	21%
FEV_1_ (L)	2.50	0.91	36%	1.19	48%	30%
FEV_1_/FVC (%)	84	64	76%	69	82%	8%
FEF 25-75% (L/S)	2.82	0.50	18%	0.69	24%	39%
FEF 50% (L/S)	4.12	0.63	15%	0.95	23%	50%
PEF (L/S)	6.40	2.07	32%	3.21	50%	55%

**Table 2:** The patient's pulmonary function testing showing severe air trapping. Residual volume is severely elevated at 274% of predicted TLC: total lung capacity; RV: residual volume; FRCpleth: functional residual capacity; VC: vital capacity; IC: inspiratory capacity; ERV: expiratory reserved volume

Lung volumes	Predicted (reference)	Actual	%Predicted	Post bronchodilator	Post-predicted%
TLC (L)	4.86	4.80	99%	-	-
RV (L)	1.15	3.16	274%	-	-
RV/TLC (%)	24	66	279%	-	-
FRCpleth (L)	2.40	3.52	147%	-	-
VC (L)	3.72	1.43	38%	1.72	46%
IC (L)	2.44	1.28	53%	-	-
ERV (L)	1.16	0.36	31%	-	-

Follow-up revealed ineligibility for bronchoscopic lung volume reduction due to ongoing cannabis smoking. Pulmonary rehabilitation and six months of smoking abstinence for a reassessment of candidacy were recommended. At five-month follow-up, there were no signs of infectious bullae, and the bullous emphysema had improved, leaving only a unilateral massive bulla in the left upper lobe. At the time of publication, she is awaiting surgery.

## Discussion

**Figure 2:**
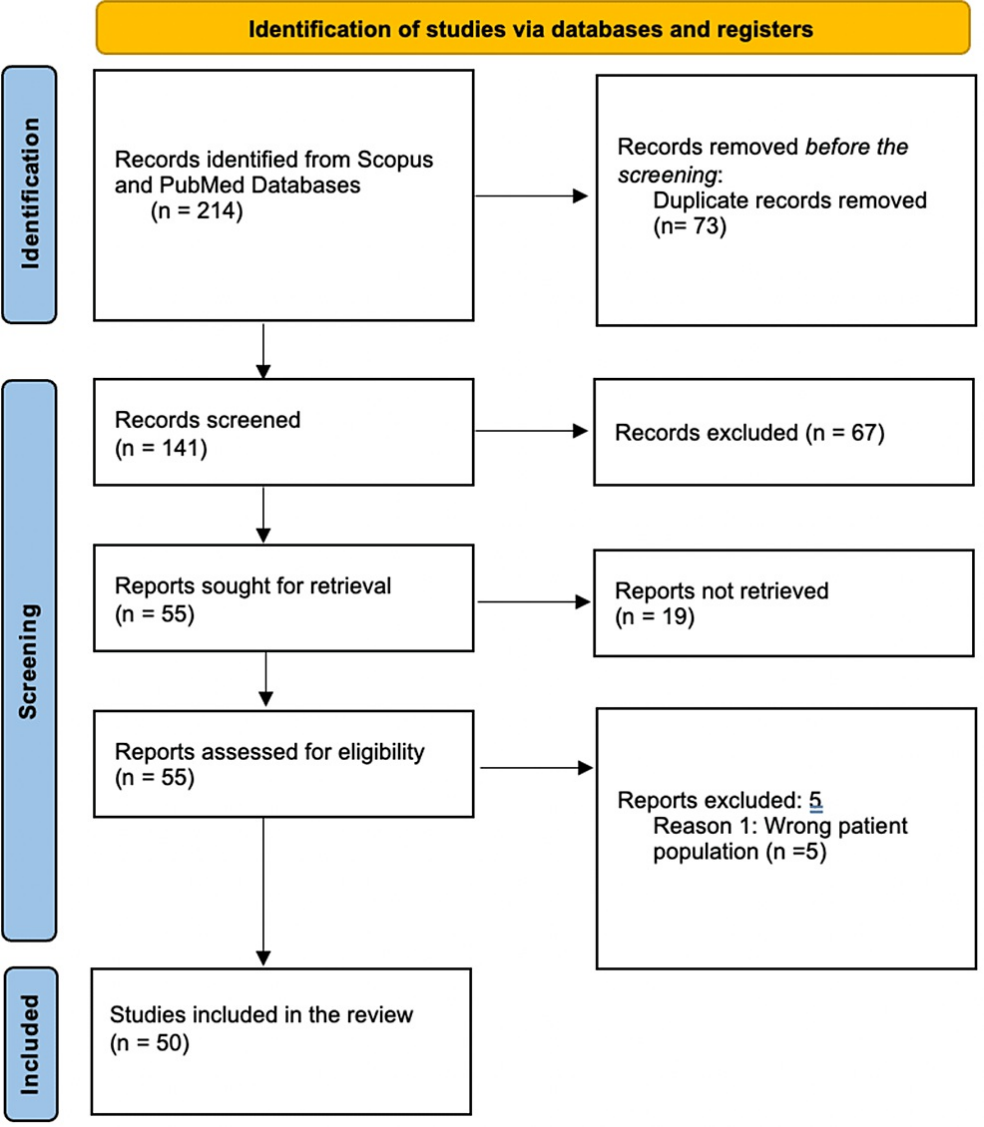
Article selection reported via the PRISMA 2020 diagram PRISMA: Preferred Reporting Items for Systematic Reviews and Meta-Analyses

In the period between September and October 2013, a search of two databases, PubMed and Scopus, identified 213 articles. Seventy-three duplicate studies were removed. After following the strict inclusion criteria of only adult patients with VLS and excluding the pediatric age group and patients with emphysema without VLS, 91 studies were excluded. The final 50 reviewed studies reported 56 patients with VLS (Figure 2). The 2009 Oxford Levels of Evidence criteria established the articles' quality of evidence (Table 3).

**Table 3:** Quality of evidence as established by the 2009 Oxford Levels of Evidence Criteria

Author	Level of evidence
Gao et al. [5]	4
Talwar et al. [7]	4
Anile et al. [8]	4
Darlong et al. [9]	4
Ye et al. [10]	4
Fila et al. [11]	4
Mani et al. [12]	4
Malhotra et al. [13]	4
Chen et al. [14]	4
An et al. [15]	4
Ye et al. [16]	4
Aujayeb [17]	4
Pekša et al. [18]	4
Wang and Liu [19]	4
Saravu et al. [20]	4
Muhamad et al. [21]	4
Jha et al. [22]	4
Miller [23]	4
Dell'Amore et al. [24]	4
Lopes et al. [25]	4
Van Bael et al. [26]	4
Gallegos and Jenks [27]	4
Giller et al. [28]	4
Lai et al. [29]	4
Hadidi and Shastri [30]	4
Piao et al. [31]	4
Ladizinski and Sankey [32]	4
Dell'Amore et al. [33]	4
Tsao and Lee [34]	4
Hossain et al. [35]	4
Luks et al. [36]	4
Ballay et al. [37]	4
Giller Dmitry et al. 2020 [38]	4
Vij et al. [39]	4
Fei and Marill [40]	4
Lin et al. [41]	4
Garvey et al. [42]	4
Satoh et al. [43]	4
Im et al. [44]	4
Saeed and Gray [45]	4
Sohail et al. [46]	4
Tashtoush et al. [47]	4
Huang et al. [48]	4
Yousaf et al. [49]	4
Navarro-Esteva et al. [50]	4
Sood and Sood [51]	4
Davies and Bradley [52]	4
MacNee [53]	4
Salley et al. [54]	4
Wiesel et al. [55]	4
West et al. [56]	4

The average age at presentation was 42.8 years, with a male predominance at 75%. Half of the patients had a prior history of lung diseases, as outlined in Table 2. Family history was unreported for 78.6% of the patients, but when mentioned, half had a family history of pulmonary disease. Notably, one report identified VLS in five family members, suggesting a potential genetic predisposition to the condition [56]. While an association between alpha-1-antitrypsin deficiency and VLS has been reported, of the 33.9% who had undergone testing, none of the patients in this study had this deficiency (Table 4).

**Table 4:** Clinical characteristics and demographics of patients with vanishing lung syndrome *: including COPD, pneumothorax, cystic fibrosis, asthma, pulmonary embolism, pulmonary sarcoidosis, obstructive sleep apnea, and lung cancer **: including VATS, thoracotomy, lobectomy, bullectomy, and pneumonectomy, unspecified CT: computed tomography; COPD: chronic obstructive pulmonary disease; VATS: video-assisted thoracoscopic surgery

Clinical characteristics and demographics of the patients in the reviewed studies	n=56	%
Gender		
Male	42	75
Female	14	25
Average age (years)	42.8	
Laterality		
Right	16	28.6
Left	15	26.8
Bilateral	20	35.7
Not reported	5	8.9
Prior lung disease*		
Yes	23	41.1
No	28	50
Not reported	5	8.9
Additional comorbid conditions		
Yes	14	25
No	40	71.4
Not reported	2	3.6
Family history of pulmonary disease		
Yes	6	10.7
No	6	10.7
Not reported	44	78.6
History of tobacco use		
Yes	38	67.9
No	15	26.8
Not reported	3	5.4
Average number of pack-years (range: 5-75 years)	30	
Cannabis use		
Yes	8	14.3
No	11	19.6
Not reported	37	66.1
Associated diagnoses found simultaneously		
Yes	14	25
No	37	66.1
Not reported	5	8.9
Treated for incorrectly diagnosed pneumothorax		
Yes	7	12.5
No	47	8.4
Not reported	2	3.6
Alpha-1-antitrypsin deficiency		
Positive	0	0
Negative	19	33.9
Not measured	37	66.1
CT used in diagnosis		
Yes	53	94.6
No	3	5.4
Not reported	0	0
Treatment		
Conservatively	9	16.1
Surgery**	30	53.6
Transplant	1	1.8
None	14	25
Not reported	2	3.6
Complications		
Yes	9	16.1
No	30	53.6
Not reported	17	30.4
Outcome		
Disease-free, alive	32	57.1
Disease persistent	5	8.9
Disease recurrence	0	0
Deceased	3	5.4
Not reported	16	2.9
Average follow-up time in months (range: 0.25-240)	70.4	

VLS is commonly misdiagnosed as pneumothorax on chest X-rays. Clinically, VLS has a more gradual onset of symptoms than pneumothorax [56]. Radiologically, visualizing the outer aspect of the visceral pleura, known as the "pleural line," separated from the parietal pleura is characteristic of pneumothorax and absent in the presence of giant bullae. A CT is typically warranted to further delineate the lung parenchyma. The characteristic appearance is subpleural bullae with surrounding paraseptal and centrilobular emphysema. It is not uncommon for giant lung bullae to coexist with pneumothorax; it is distinguished on CT by air on both sides of the bullae wall, called the "double-wall sign" [57,58]. Per review, CT was used to establish a diagnosis of VLS in 94.6% of cases. Treatment was initiated in seven patients based on an initially incorrect diagnosis of pneumothorax, but these cases were eventually corrected. Similarly, our patient initially presented with suspected pneumothorax in the setting of a COVID-19 infection; however, subsequent CT suggested VLS.

Management of VLS patients includes lifestyle modifications, smoking cessation, and influenza and pneumococcal vaccination. Nebulized bronchodilators and inhaled corticosteroids are also frequently used [44]. In this review, 67.9% of patients smoked tobacco, with an average of 30 pack-years. Cannabis use was reported in 14.3% of patients. Of the seven patients who had a history of cannabis use, five patients (71%) used cannabis and tobacco concurrently. Our patient also reported tobacco and cannabis use and was deferred from surgery until abstinent.

Surgical intervention is typically indicated for symptomatic VLS patients. Determining surgical candidacy involves considering various factors, including the patient's age, pulmonary function testing results, body mass index, and overall quality of life [44]. Thoracoscopic versus open bullectomy is the gold standard surgical approach. Additional surgical options involve lung volume reduction surgery (LVRS) in the presence of severe coexisting emphysema. A one-way endo-bronchial valve can be deployed to mitigate air trapping and reduce bullae size. In rare cases, lung transplantation is necessary [44,59]. A majority (53.6%) of patients in this review were managed with surgery with reported symptomatic improvement in shortness of breath, functional activity, and frequency of hospitalizations. While our patient was initially under conservative management, the increasing size of the bullae prompted a referral for lung volume reduction surgery.

## Conclusions

This case and review highlight the diagnostic complexities associated with VLS, emphasizing the need to consider diverse demographic factors and risk associations, use appropriate diagnostic tools, and make informed treatment recommendations. Our literature search found that VLS was more predominant in males and that most patients who develop VLS have a predisposing lung disease such as asthma, COPD, sarcoidosis, or obstructive sleep apnea. Our review also sheds light on VLS mimicking pneumothorax radiologically. Clinicians should have a high index of suspicion to differentiate between pneumothorax and VLS, as it can affect patients' clinical course and treatment plan. Symptomatic VLS patients who are surgical candidates have a better outcome with surgical intervention compared to conservative management. Further research is needed to delineate the demographic and clinical characteristics of VLS patients in order to increase clinician's diagnostic vigilance. 
